# Comparative analysis of nanobody sequence and structure data

**DOI:** 10.1002/prot.25497

**Published:** 2018-04-15

**Authors:** Laura S. Mitchell, Lucy J. Colwell

**Affiliations:** ^1^ Department of Chemistry University of Cambridge, Lensfield Road Cambridge CB2 1EW United Kingdom

**Keywords:** antibody, camelid, framework, HcAb, heavy chain antibody, loop, single domain antibody, VH, VHH

## Abstract

Nanobodies are a class of antigen‐binding protein derived from camelids that achieve comparable binding affinities and specificities to classical antibodies, despite comprising only a single 15 kDa variable domain. Their reduced size makes them an exciting target molecule with which we can explore the molecular code that underpins binding specificity—how is such high specificity achieved? Here, we use a novel dataset of 90 nonredundant, protein‐binding nanobodies with antigen‐bound crystal structures to address this question. To provide a baseline for comparison we construct an analogous set of classical antibodies, allowing us to probe how nanobodies achieve high specificity binding with a dramatically reduced sequence space. Our analysis reveals that nanobodies do not diversify their framework region to compensate for the loss of the VL domain. In addition to the previously reported increase in H3 loop length, we find that nanobodies create diversity by drawing their paratope regions from a significantly larger set of aligned sequence positions, and by exhibiting greater structural variation in their H1 and H2 loops.

## 1 INTRODUCTION

The efficacy of an immune system directly reflects the diversity of antigens against which specific, tightly binding B‐lymphocyte antigen receptors (BCRs) can be generated. Conventional full‐length antibodies (Abs) have become essential tools in biological research, and a foundation of the biopharmaceutical industry due to their exquisite binding specificity and high affinity to target antigens. The immense diversity of binding specificity is created by sequence variation in two variable domains, the heavy chain (VH) and the light chain (VL). Together, these have been estimated to yield a diversity of at least 10^15^ possible BCRs in humans,^1^, ^2^, ^3^ easily exceeding the B‐lymphocyte population size in an individual (∼ 10^11^).^4^ Exactly how the enormous potential sequence diversity translates into antigen specificity is not known. It is clear that there is some redundancy—not every sequence unique VH‐VL combination results in a unique binding specificity. However, the number and locations of amino acid mutations required to change binding specificity have proved difficult to predict.^5^, ^6^

**Figure 1:**
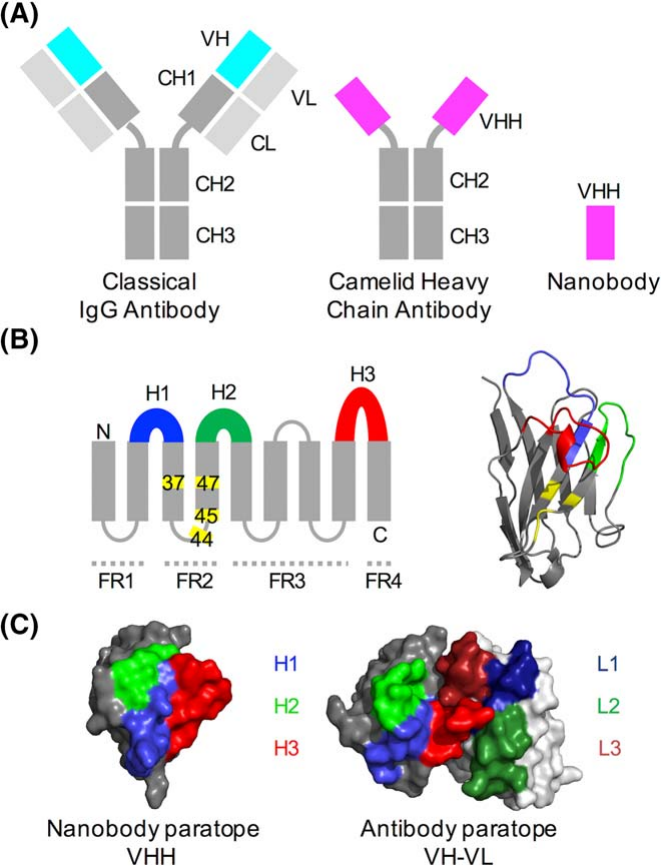
Structural features of conventional and camelid heavy‐chain Abs. A, In an Ab, the antigen binds to the VH‐VL interface, while in the camelid heavy‐chain antibody the VH‐homologous VHH domain binds the antigen. Nbs are produced when the VHH domain is expressed in bacterial systems. In Abs, the VH and VL domains bind to each other and can only be produced in bacterial expression systems when joined by a peptide linker. B, Like the Ab VH domain, the secondary structure of the Nb VHH domain consists of 9 beta sheets separated by loop regions, 3 of which are hypervariable (shown in blue, green and red). Four framework regions (FRs) separate the variable loops; these are less sequence‐variable. Four positions known as the VHH‐tetrad are numbered and highlighted in yellow. Right: VHH domain with VHH‐tetrad positions in yellow. C, The antigen‐binding surface in Nb VHH domains and Ab VH‐VL domains (aligned orientations) [Color figure can be viewed at http://wileyonlinelibrary.com]

A potentially more tractable system is provided by the class of heavy‐chain antibodies found in camelid species such as camels, llamas and alpacas, for which the light chain is completely absent (Figure 1). The ≈ 15 kDa isolated variable VHH domain, known as a nanobody (Nb), is approximately ten times smaller than a conventional Ab yet retains comparable binding specificity.^7^ Nbs exhibit improved stability and are able to bind Ab‐inaccessible epitopes in enzyme active sites, viral capsids and G protein coupled receptors.^8^, ^9^, ^10^, ^11^, ^12^ The camelid VHH domain that forms the Nb is homologous to the Ab VH domain and contains three highly variable loops H1, H2, and H3 (Figure 1). These loops form an extended structural interface at one side of the folded protein domain that contributes to the antigen‐binding interface, or paratope, which determines the Nb antigen‐binding specificity.^7^ The potential sequence diversity of these three loops is much smaller than that of the six highly variable loops that largely determine the binding specificity on the Ab VH‐VL domain complex.^2^, ^13^, ^14^, ^15^

For both Nbs and Abs, the fundamental challenge is to determine the molecular code that relates amino acid sequence, and in particular choice of paratope residues, to the binding specificity of the folded molecule.^16^ In conventional Abs, the paratope lies at the interface of the VH and VL domains and typically contains residues from as many as six distinct hypervariable loop regions.^17^ There is also considerable freedom in how the VH‐ and VL‐ domains dock together, allowing the Ab to maximize the diversity of possible antigen‐binding surfaces.^13^ In contrast, the Nb paratope is entirely contained within the VHH domain (Figure 1C), drastically reducing the space of possible antigen‐binding surfaces without apparently affecting the diversity of resulting binding specificities.^10^ Indeed, Nbs typically bind their target antigen with affinities comparable to those achieved by classical monoclonal Abs.^2^ How can Nbs generate such a diversity of binding specificities despite their small size and single domain architecture?

To address this question, we compile a dataset of 90 nonredundant protein binding Nbs for which Nb‐antigen co‐crystal X‐ray structures are available, allowing us to identify and study a diverse set of Nb paratopes. Early studies examined sequence and structural diversity in the hypervariable loop regions of small sets of Nbs.^2^, ^10^, ^18^, ^19^, ^20^ The sequence and structure dataset that we compile allows us to examine the Nb paratope in detail. The collated structures span a diverse set of 72 distinct epitopes on antigens that include membrane proteins, viral proteins, enzymes, and intrinsically disordered proteins. To facilitate comparison with classical Abs we collate a comparable dataset containing 90 nonredundant protein binding Abs for which Ab‐antigen co‐crystal X‐ray structures are available. We use structural alignments to compare sequence and structure variation within the variable loops and framework regions of Nb VHH domains and Ab VH domains. These datasets enable us to provide the first comprehensive description of the sequence and structural variability present in Nb VHH domains, and to examine how this variability is exploited to generate distinct binding specificities.

## 2 MATERIALS AND METHODS

### 2.1 Data collection

We built two datasets of co‐crystal protein complex structures, each containing a Nb‐antigen or Ab‐antigen protein complex. Our novel dataset consists of 90 nonredundant protein binding Nbs for which Nb‐antigen co‐crystal structures are available in the PDB.^21^ To construct this dataset all crystal structures containing protein‐bound Nbs were downloaded from the PDB, and filtered using a 100% sequence identity threshold to give rise to a set of sequence‐unique Nb structures. No crystal structure resolution threshold was applied, to maximize the number of structures in the dataset. The structural resolutions range between 1.1 and 4.1 Å, with a mean of 2.2 Å. Of the 90 Nbs, 60 derive from *Lama glama* (llama), 24 from *Camelus dromedarius* (camel), and 6 from *Vicugna pacos* (alpaca). The Nbs in the dataset bind to 72 structurally unique epitopes, where a unique epitope is defined as having <50% of epitope residues in common with another epitope in the dataset. The set of 90 Ab‐antigen complex structures were chosen at random from those used in a recent study.^22^ The Ab dataset contains 90 sequence distinct VH domains, which bind 75 structurally unique epitopes, with resolutions that range 1.2–2.8 Å, with a mean of 2.3 Å.

### 2.2 Sequence alignment

**Figure 2:**
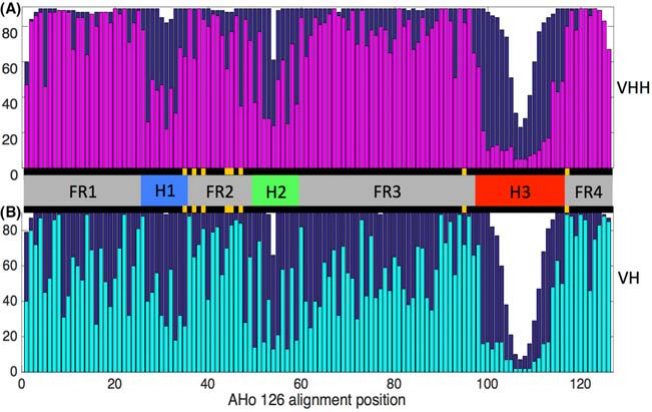
A, Summary of sequence variability in sets of Nb VHH domain and B, Ab VH domain sequences. The number of sequences with a residue (ie, not a gap character) at each alignment position is plotted in dark blue. The number of sequences with the most frequent amino acid at that position (nWT) is overlaid in magenta or cyan. The central horizontal bar indicates the framework (gray) and loop regions (colored). Despite comparable diversity of epitope specificities among the two datasets we note that the framework regions of the Nb VHH domains are significantly more conserved than those of the Ab VH domains. Those alignment positions that are involved in the VH‐VL interface, and the equivalent positions in VHH domains, are marked in yellow [Color figure can be viewed at http://wileyonlinelibrary.com]

**Figure 3:**
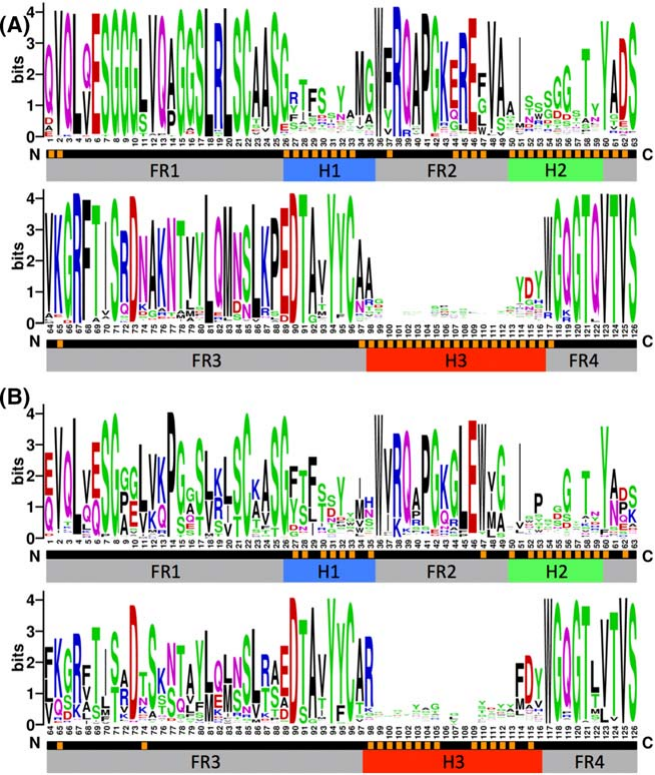
A, Sequence logo plots that show the amino acid variation present in the Nb VHH domains and B, Ab VH domains from the datasets described. Aligned sequence positions with high propensity (> 10% of structures) to contact the antigen are marked in orange. Note the difference in residue composition between VH and VHH at the four VHH‐tetrad positions 37, 44, 45, and 47. Our alignments show VHH position 47 can frequently be Phe, Leu or Trp in addition to the often cited Gly. It is also interesting that the two VHH charged residues at positions 44 and 45 are frequently in contact with the antigen, rather than with the VL, as they are in VH‐VL complexes [Color figure can be viewed at http://wileyonlinelibrary.com]

Sequences of Nb VHH domains were extracted from the PDB files and aligned using the online tool ANARCI^23^ with the AHo alignment scheme,^24^ which arranges the residues within each loop symmetrically from the outside toward the center of each loop. This results in alignments where sequence positions toward the center of each loop contain aligned amino acids in only a few sequences. For the visualization of alignment statistics in Figures 2 and 3, alignment positions with >85% gaps were removed, reducing our alignment to just 126 positions.

**Figure 4:**
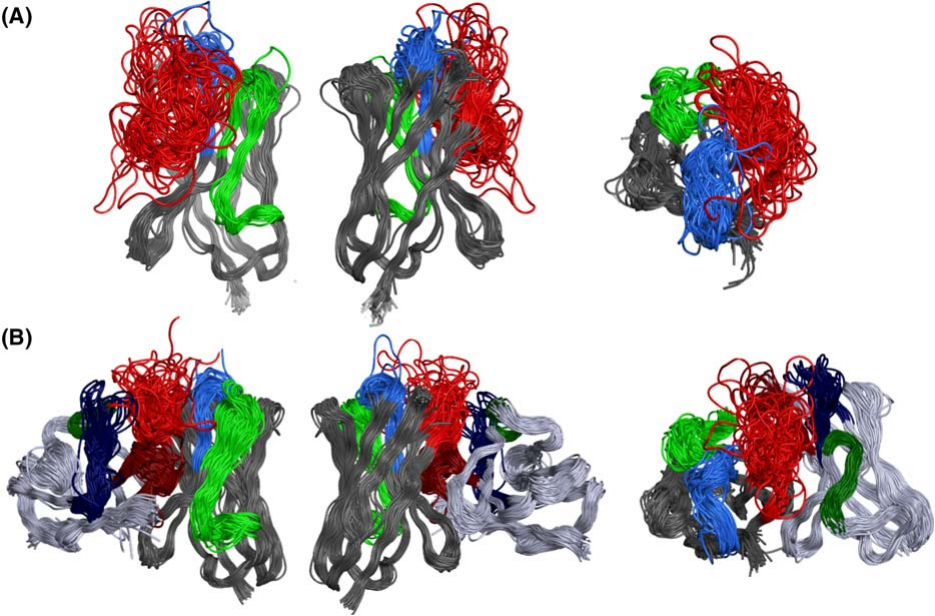
Comparison of Nb structural diversity to Ab structural diversity. Superposition of A, 90 Nb VHH domains and B, 90 Ab VH‐VL domains from co‐crystal structures. Front, back and plan views, with H1 in blue; H2 in green and H3 in red. The VHH and VH domains are aligned in each of the three views shown

**Figure 5:**
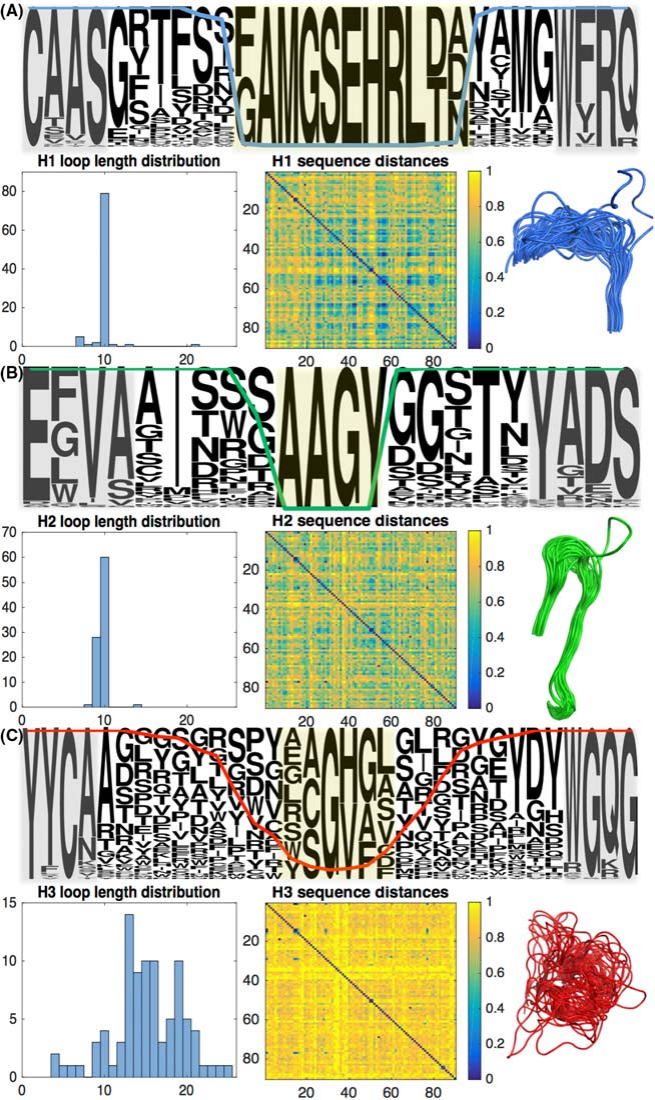
Sequence and structural analysis of Nb hypervariable loops H1–3. We characterize amino acid usage, loop length distribution, sequence diversity and structural diversity of Nb A, H1 loops, B, H2 loops, and C, H3 loops. Central loop positions with more than 85% aligned gap characters, which were excluded from the 126‐position alignment, are highlighted in yellow. Framework “anchors,” highlighted in gray, can be used to detect the loop locations in individual sequences^19^ [Color figure can be viewed at http://wileyonlinelibrary.com]

**Figure 6:**
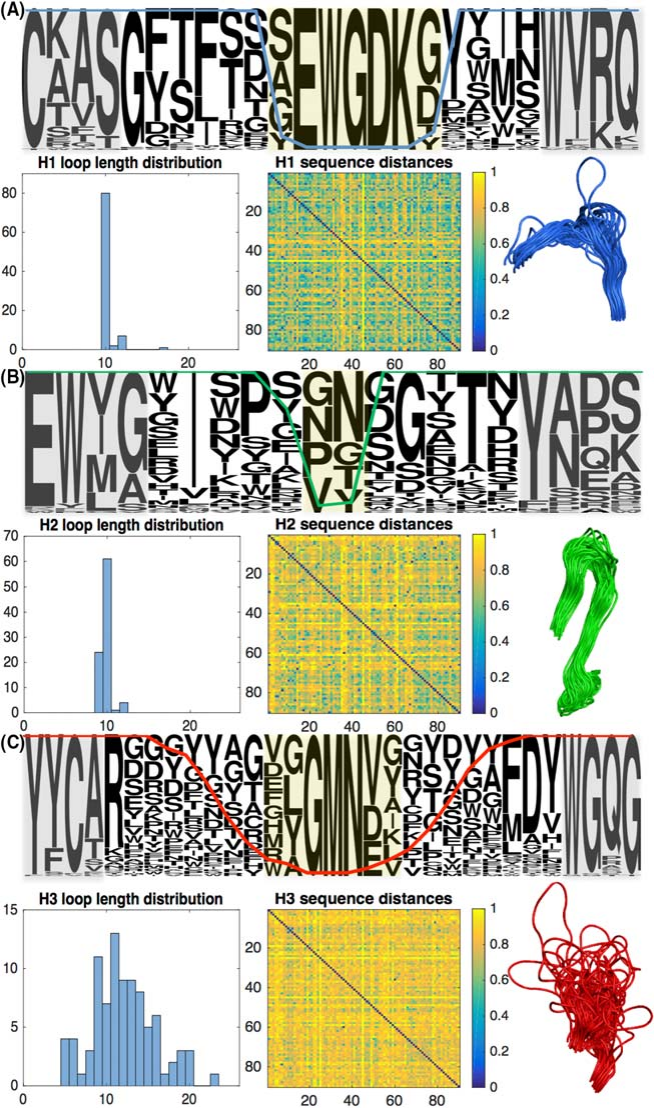
Sequence and structural analysis of Ab VH domain hypervariable loops H1–3. We characterize amino acid usage, loop length distribution, sequence diversity and structural diversity of Ab A, H1 loops, B, H2 loops, and C, H3 loops. Central loop positions with more than 85% aligned gap characters, which were excluded from the 126‐position alignment, are highlighted in yellow. Framework “anchors,” shown in gray, are more variable than in the VHH alignment, but can still be used to detect the loop locations in individual sequences [Color figure can be viewed at http://wileyonlinelibrary.com]

Similarly, sequences of Ab VH domains were extracted from the 90 PDB files contained in our dataset and aligned using the same approach. In the resulting alignments the distinction between framework and hypervariable loop regions were clear, and concurred with visual inspection of the 3D structures, shown in Figure 4. Furthermore for the Nb sequence alignment, our alignment reproduced the hypervariable loop “anchors” proposed previously.^19^ To retain information about central loop position variability the original full length alignments were used for loop analysis in Figures 5 and 6. Antigen‐bound Nb and Ab PDB and chain IDs, and apo‐form Nb PDB and chain IDs are listed in Supporting Information Tables S1–S3, respectively.

Throughout this work, we refer to amino acids by their aligned sequence positions. In our alignments, the variable loops H1–3 are positions 26–35, 50–59, and 98–116 for both Nbs and Ab VH domains. These loop definitions are based on those used by Sircar et al.^19^ but modified to include two further positions at the C‐terminus of the H2, and one additional position at the N‐terminus of the H3. This ensures all sequence and structure‐variable positions are included in our analysis of loop variability. AHo is included in Supporting Information Table S4.

### 2.3 Sequence logos

All sequence logos were generated by submitting the relevant sequence alignments to the WebLogo server Version 2.8.2.^25^ The logos in Figure 3 plot the sequence conservation for each position as the height of the stack in bits, a measure of information content. The height of each residue within a stack indicates the relative frequencies of each amino acid at that position. The logos in Figures 5 and 6 plot residue frequency rather than information content, because this representation more clearly shows the residue usage across central positions in longer loops. The Hamming distance between two aligned sequences counts the number of positions at which the sequences differ, normalized by sequence length to give a score between 0 and 1.

### 2.4 Structure alignment and identification of antigen contacting residues

The antigen‐bound Nb and Ab structures were then used to build a global structural alignment for each dataset, shown in Figure 4, using the superposition function in MOE 2015.1001.^26^ For the Ab dataset, we aligned the part of the molecule consisting of both the VH and VL domains to reflect the complete structural unit involved in molecular recognition and antigen binding, as it is known that the angle between the VH‐ and VL‐domains can vary significantly.^13^ For the Nb dataset, we aligned the structures over the VHH domains. To identify antigen‐contacting residues from the two sets of structures, we identify all residues in each co‐crystal structure for which the minimum atom distance to the nearest antigen amino acid is <5 Å. Those residues that contact the antigen in > 10% of structures are marked on the sequence alignment in Figure 3 in yellow.

To quantify the structural variability of different sections of the Nb VHH domain and the Ab VH domain we calculated the average pairwise root mean square deviation (RMSD) among each set of structures over the residue sets indicated in Tables 1 and 2. We first extracted the framework region C‐α atomic coordinates from each PDB file, and then computed the optimal structural alignment between every pair of dataset structures using the Procrustes or Kabsch algorithm.^27^ In each case, the pairwise RMSD for each structural subunit was then calculated over those aligned sequence positions for which both structures had PDB coordinates. Statistics describing the number of aligned positions for each loop are reported in Table 2. Note this calculation omits the few central loop residues not included in the 126‐position alignment (marked yellow in Supporting Information Figures S1 and S2). This affects two Nb H1 loops, one Nb H2 loop and twelve Nb H3 loops, in addition to eleven Ab H1 loops, six Ab H2 loops, and four Ab H3 loops (see Figures 5 and 6 for loop length distributions).

**Table 1:** Mean and standard deviation of the pairwise RMSDs calculated over the 87 aligned C‐α atoms contained in framework regions 1–4, for the variable domain structures from each dataset

Dataset	RMSD over VHH or VH domain framework regions 1–4
90 Nb VHH domains, antigen‐bound	1.01 ± 0.31 Å
90 Ab VH‐VL units, antigen‐bound	1.39 ± 0.51 Å
90 Ab VH domains, antigen‐bound	1.12 ± 0.50 Å
58 Nb VHH domains, apo form	0.95 ± 0.28 Å

In the case of the Ab VH‐VL units we carried out the alignment using two approaches: first, we aligned over all framework C‐α atoms from both the VH and VL domains and secondly, we aligned over just the framework C‐α atoms from just the VH domain. The second approach results in a lower RMSD.

**Table 2:** Mean and standard deviation of the pairwise RMSDs calculated over all pairs of sequence‐aligned C‐α atoms contained in the H1–3 loop segments, for the variable domain structures from each dataset

Dataset	H1 loop	H2 loop	H3 loop
90 Nb VHH domains, antigen‐bound	3.36 ± 1.11 Å 9.93 ± 0.37 positions	1.86 ± 0.74 Å 9.54 ± 0.54 positions	6.43 ± 3.32 Å 11.46 ± 3.57 positions
90 Ab VH domains, antigen‐bound	1.60 ± 0.92 Å 10.00 ± 0.23 positions	1.78 ± 0.70 Å 9.52 ± 0.55 positions	3.52 ± 1.33 Å 9.95 ± 2.86 positions
58 Nb VHH domains, apo form	3.28 ± 1.24 Å 9.59 ± 1.00 positions	1.93 ± 0.70 Å 9.37 ± 0.59 positions	6.28 ± 2.89 Å 12.82 ± 3.71 positions

The RMSDs reported for H3 loops should be interpreted carefully because the high degree of structural diversity over this segment has the effect that pairs of sequence‐aligned positions are often not structurally comparable. The difference in mean pairwise RMSD between the antigen bound Nb and Ab datasets for each loop is statistically significant according to a two‐tailed unpaired *t* test at the 0.05 significance level.

## 3 RESULTS AND DISCUSSION

To investigate and compare the molecular code used by Nbs and Abs to generate diverse binding specificities we identified 90 co‐crystal structures for both Nb‐antigen and Ab‐antigen complexes. The set of 90 Nb complexes contains 72 distinct structural epitopes, while the set of 90 Ab‐antigen complexes contains 75 distinct structural epitopes. Using this data we analyze Nb and Ab structural variation in the antigen bound conformation.

These data allow us to address the key question of how Nbs generate such a diverse range of binding specificities despite their reduced sequence length and compact single domain architecture. Much work has shown that the six hypervariable loops shown in Figure 1C are key to determining Ab interaction specificity.^6^, ^22^, ^28^, ^29^ Nbs, in contrast, have only three hypervariable loops, reducing the space of possible sequence variants and hence the potential interaction specificities. This could be compensated in a number of ways, for example: (i) increasing the length of the three variable loops,^19^, ^30^ (ii) increasing the level of sequence variation outside the variable loops, and (iii) increasing the diversity of amino acids within the loop regions. Here we use our assembled data to address this question.

### 3.1 Nanobody sequence analysis

In Figure 2, we use the compiled data to compare Nb VHH domain and Ab VH domain sequence variability. Sequences were extracted from the set of PDB files and aligned using a structure based protocol (see methods), allowing the framework and hypervariable loop regions of each alignment to be identified as shown in Figure 2. The number of aligned, un‐gapped sequences at each position of the Nb (Figure 2A) and Ab (Figure 2B) alignments are plotted in dark blue. To confirm that these alignments are representative, we apply the same analysis to larger sets of Nb and Ab sequences in Supporting Information Figure S3. Positions where insertions or deletions tend to occur are mainly found in hypervariable loop regions H1–3.^19^, ^20^, ^31^ To visualize the sequence conservation at each aligned position, we plot the number of sequences with the most prevalent (or wild type) amino acid in magenta (Nb alignment, Figure 2A) and in cyan (Ab alignment, Figure 2B). Both alignments display high levels of variability within the three loop regions H1–3. Perhaps surprisingly, Figure 2 shows that overall the Nb framework is much more conserved than that of the Ab VH domains.

It is important to verify that the framework conservation shown in Figure 2A is not merely an artifact of the small set of camelid species that produce Nbs. Species specific sequence and structural properties of classical Abs have previously been reported.^32^ To provide a control, we assembled large single‐species (mouse VH domain and human gamma1 VH domain) Ab sequence alignments, and large single‐species Nb VHH domain sequence alignments for *Lama glama, Vicugna pacos*, and *Camelus dromedaries* from the abYsis database.^33^ From each single‐species alignment we randomly draw subsets of 90 sequences, and plot the resulting species‐specific conservation profiles in Supporting Information Figure S4. We find that subsets of 90 Ab VH domains drawn from single species show much greater sequence diversity than even the multi‐species Nb alignment shown in Figure 2A. The greater Ab diversity likely reflects the availability of many V gene families (*IGHV1*, *IGHV2*, and so forth) that give rise to Abs, compared to the smaller number of V gene segments in *IGHVH* that are used to produce the majority of Nbs.^7^, ^34^

The striking finding revealed by Figure 2 is that the Nb VHH domain framework is significantly more conserved than the Ab VH domain framework. Overall, we find that > 70% (89/126) Nb sequence positions (73% of FR positions, 64/87) have a significantly different level of conservation to the equivalent Ab positions, that is, ±2σ from the mean Ab position specific conservation level from 6 bootstrapped alignments (see Supporting Information Table S5 for details). Only 7/87 Nb framework positions—14, 37, 44, 45, 47, 92 and 117 are significantly more variable than the corresponding Ab positions. Five of these positions are found on the VH‐VL interface (yellow in Figure 2), and are conserved in VH domains to maintain VH‐VL pairing. Greater variability in Nbs at these positions reflects that they are not under this evolutionary constraint, so could contribute to specificity for example by altering H3 loop packing against the domain.

The greater framework conservation in Nbs is surprising; as Nbs might be expected to increase their sequence variation, to compensate for the reduced potential sequence diversity due to their small size. Figure 2 suggests that this is not the case, a conclusion supported by sequence logos generated for each alignment (Figure 3), which also compare amino acid usage at each site. Similarities are largely limited to amino acid usage at the C‐termini, while there are multiple distinct differences in residue usage, in particular among the eight VH‐VL interface positions. Here, Ab VH domains contain networks of hydrophobic residues (positions V37, G44, L45, W47, Y95, and W117), thought to be required for maintaining pairing with the cognate VL‐domain.^20^ In Nb VHH domains G44E and L45R reduce hydrophobicity, while V37F and W47F/G are often shielded from solvent by the H3 loop.^7^, ^34^, ^35^ Our data support sequence differences reported in previous studies.^19^, ^20^ In addition Figure 3 shows that throughout the FR3 region Nbs make greater use of charged residues. Specifically D62, K65, R67, R72, K76, and E89 are all more prevalent in Nb VHH domains compared to Ab VH domains, increasing the solubility of the isolated VHH domain.

Our datasets of co‐crystal structures allow us to identify residues that lie on the interface with the protein antigen—that is, the paratope (see methods). These sequence positions play an important role in determining interaction specificity. Overall, the average number of antigen‐contacting residues is 18.76 per VHH structure, compared to 16.01 per VH structure and 24.91 per VH‐VL unit.

In Figure 3, we highlight those sequence positions that lie in > 10% of dataset paratopes in yellow. Paratope positions are largely contained within the three hypervariable loop regions for both Nbs and Ab VH domains. However, while Ab paratopes are drawn from just 35 aligned positions, Nb paratopes are more diverse, with 50 aligned sequence positions appearing in > 10% of the 90 dataset paratopes, as shown in Figure 3. This suggests that Nbs generate diverse specificities by drawing their paratopes from a larger set of aligned sequence positions. Five of the additional positions used by Nbs are found in the H3 loop, reported to be longer in Nb VHH domains.^7^, ^10^, ^19^, ^30^ The additional paratope positions include multiple framework residues located at the N‐terminus, across the FR2 region, and adjacent to the hypervariable loops. Furthermore, Figure 3 shows that paratope positions tend to be less conserved than neighboring nonparatope framework positions, as might be expected if they help determine interaction specificity.

### 3.2 Structural variation of framework regions

Structural variation of the framework region is another potential source of Nb diversity. To examine this we built structural alignments of Nb VHH domains, Ab VH domains, and Ab VH‐VL complexes. Figure 4 shows alignments of the 90 antigen bound Nb structures (Figure 4A) and the 90 antigen bound Ab VH‐VL domain structures (Figure 4B) built using the align‐all function in MOE^26^ (see methods). The three hypervariable loops of the VHH domain are colored with loop H1 in blue, H2 in green and H3 in red. In Figure 4B, the variable loops of Ab VH domain use the same color scheme, while those of the cognate Ab VL‐domain are colored using a darker version thereof. To gain insight into the impact that antigen binding has on structural variability, we also include an alignment of the 58 Nbs for which crystal structures are available in the apo form in Supporting Information Figure S5.

To measure the structural diversity present in these datasets we first extracted the C‐α atomic coordinates corresponding to the framework regions. We then used the Procrustes (Kabsch) algorithm to find the linear transformation resulting in the optimal alignment for each pair of crystal structures and built the set of all pairwise alignments for each dataset.^27^ This allowed us to compute the matrix of pairwise RMSD values for each dataset—the Nb VHH domains, the Ab VH domains, the Ab VH‐VL domain complexes and also the apo form Nb VHH domains. The mean RMSD values across the framework regions calculated from these matrices are reported in Table 1.

We find that the 90 Nb structures align relatively tightly over the framework regions, with an average pairwise RMSD of 1.01 ± 0.31 Å measured over the C‐α atoms of the 87 aligned framework residues. The equivalent value for the 58 apo form Nbs is similar to this, at 0.95 ± 0.28 Å. In contrast, the 90 Ab VH domains have an average pairwise RMSD of 1.12 ± 0.50 Å measured over the 87 framework C‐α atoms. This RMSD increases to 1.39 ± 0.51 Å if the structural alignment is carried out over the FRs from both VH and VL domains. This suggests that Nbs display a slight yet significant (two‐tailed unpaired *t* test, *P* = 3.14 E –33) reduction in framework structural variability compared to Ab VH domains, allowing us to conclude that Nbs do not increase diversity by varying their FR structure.

### 3.3 Nanobody loop analysis

Our analysis so far suggests that any additional mechanism for generating Nb interaction specificity must be contained in the three hypervariable loops H1–3. Indeed, Figure 2A suggests that in contrast to the framework regions, Nb loops are at least as sequence variable as Ab VH domain loops. Furthermore, Figure 4A shows that the greatest structural variability is found in the Nb H3 loop (shown in red). Previous work has found that Nbs possess particularly long H3 loops,^7^, ^10^, ^19^, ^30^ which could potentially greatly increase both the sequence and shape diversity of the Nb paratope.

Figure 5 presents analysis of the full‐length Nb VHH domain loops H1–3 in our dataset, which contains 82, 83, and 86 sequence‐unique H1, H2, and H3 Nb loops, respectively. For each loop, we construct a sequence logo; a histogram showing the distribution of loop lengths; a heat map showing the normalized Hamming distance that is, fraction of amino acid differences between every pair of aligned loops; and the extracted section of the structural alignment of Figure 4. Within the logo plots, gray boxes indicate the framework regions immediately adjacent to each loop, which act as sequence flags enabling the loops to be located in the absence of structural information.^19^ Those sequence positions at the center of each loop with > 85% gap characters are shaded in yellow and excluded from the sequence alignments analyzed in Figures 2 and 3. The percentage of nongap characters at each position is superimposed onto each sequence logo plot as a colored line. For comparison we present the analogous analysis of the Ab VH domain dataset in Figure 6. Our Ab dataset contains 84, 86, and 89 sequence‐unique H1, H2, and H3 Ab VH domain loops respectively. The RMSDs calculated for loop segments H1–3 of VHH domain and VH domain structural alignments are reported in Table 2.

We focus first on the H1 loops. The sequence logos suggest that Nb VHH and Ab VH domain H1 loops have similar features, making use of hydroxyl‐containing Ser, Tyr and Thr residues as well as bulky hydrophobic residues such as Met and Phe. Perhaps surprisingly, we find that the lengths of both H1 and H2 loops are tightly constrained for Nbs and Abs at roughly 10 amino acids in all cases. To assess loop sequence diversity, we calculate the mean normalized Hamming distance across each loop type; the Nb H1 loops have mean 0.66 ± 0.17, which is almost identical to Ab VH domain H1 loops with mean 0.66 ± 0.18.

Our structural alignments suggest that the N‐terminal end of the Nb H1 loop is more diverse than its Ab VH domain counterpart. Overall, the average pairwise H1 loop RMSD (excluding the yellow shaded residues) is 3.36 ± 1.11 Å for Nb VHH domains compared to 1.60 ± 0.92 Å for Ab VH domains. We note that aligned positions with particularly large RMSD values are concentrated at the N‐terminal end of the H1 loop. If the alignment is instead carried out across the H1 loop itself, the average pairwise RMSD values reduce to 2.06 Å for Nb VHH domains and 1.03 Å for Ab VH domains. The larger Nb H1 RMSD values may be partly explained by the fact that the conserved Phe at position 29 in Abs (position 4 in Figures 5 and 6) is less frequently observed in Nbs. The residue F29 is thought to be a stabilizing feature of the canonical Ab Type 1 H1 loop class,^36^ so reduced prevalence in Nbs may increase structural diversity. Indeed, early studies of Nb crystal structures noted that Nb H1 loops do not fit the canonical Ab Type 1 conformation.^19^, ^37^ Furthermore, NMR studies^38^ and molecular dynamics simulations^32^ suggest the Nb H1 loop may be conformationally dynamic. The set of apo‐Nbs shown in Supporting Information Figure S5 displays similar H1 loop structural variation to the antigen‐bound Nbs with an average pairwise RMSD of 3.28 ± 1.24 Å.

Our analysis finds that Nb H2 loops are less sequence‐variable than Ab VH domain H2 loops. The mean Hamming distance is 0.69 ± 0.14, corresponding to seven out of ten differences between each pair of Nb H2 loops, compared to 0.76 ± 0.15 or nearly eight out of ten differences between each pair of Ab VH domain H2 loops. A two‐tailed unpaired *t* test at the 0.05 significance level shows this difference is statistically significant (*P* = 1.81 E –121). In contrast, the average pairwise RMSD of 1.86 ± 0.74 Å for Nb H2 loops (1.93 ± 0.70 Å for apo Nb structures) suggests slightly greater backbone structural variation than Ab H2 loops at 1.78 ± 0.70 Å RMSD. This slight but significant (*t* test, *P* = 3.42 E –6) increase in Nb H2 loop average pairwise RMSD is greater if calculated across structural alignments of H2 loops for each dataset: 1.20 Å for bound Nb VHH domains compared to 1.00 Å for Ab VH domains. This is surprising, given the decrease in Nb H2 loop sequence variation, but could reflect differences in amino acid composition: Nb H2 loops feature small residues such as Gly and Ser permitting the tight backbone turn seen in a number of Nb structures, while the Ab H2 loop shows a bias toward larger residues, see Figure 3. In conclusion, we find that Nb H1 and H2 loops are less sequence diverse than the Ab loops; but nonetheless display increased structural variation. This likely contributes to increased diversity of binding specificities.

Finally, we turn to the H3 loops. Both Nb and Ab H3 loops vary significantly in length. The mean Nb H3 loop length in our dataset is 15.19 ± 4.23 residues (median length 15), while the mean Ab H3 loop length is 12.09 ± 3.81 residues (median length 12). Loop length distributions are shown in Figures 5 and 6; the Kullback‐Leibler divergence of the Nb H3 loop length distribution from the Ab H3 loop length distribution is 0.29. Nb H3 loops are also more sequence diverse, with mean Hamming distance 0.89 ± 0.09, that is, ≈ 13/15 sequence changes between each pair of Nb H3 loops, compared to 0.83 ± 0.11 that is, ≈ 10/12 residue differences between each pair of Ab VH domain H3 loops. A two‐tailed unpaired *t* test at the 0.05 significance level shows this difference is statistically significant (*P* = 6.28 E –7). In addition, the set of Nb H3 backbone structures are notably more structurally diverse than the Ab H3 loop superposition shown in Figure 6. Calculation of the average pairwise RMSD of H3 loops reveals the extent of structural variability (see Table 2 and methods); Nb VHH H3 loops have a mean pairwise RMSD of 6.43 ± 3.32 Å, and a maximum pairwise RMSD of 21.09 Å, while Ab VH H3 loops have a mean pairwise RMSD of 3.52 ± 1.33 Å, with maximum 13.14 Å. The Nb H3 RMSD values are large enough to suggest that distance measurement of sequence‐aligned atoms makes little structural sense, since the loop backbones are so distinct. Furthermore, there are clear differences in residue usage at the N‐ and C‐terminal portions of the H3 loops. Loop position 1 is a relatively conserved Ala in Nb, but Arg in Ab. Similarly, the −3 position at the C‐terminus of the loop is often a Tyr in Nb but a Phe in Ab. One simple distinction that facilitates increased structural variation is that the Nb H3 loop can occupy a greater range of positions relative to the framework due to the absence of a cognate VL‐domain. This suggests that it might be difficult to emulate the recent progress made in structural prediction of Ab H3 loops^20^, ^39^ for Nbs.

## 4 CONCLUSION

Nbs provide a simple system with which to probe the question of how homologous amino acid sequences can encode a vast diversity of antigen binding specificities. These small single domain antibodies generate high affinity binding specificity against a huge range of protein antigens, despite being half the size of the antigen‐binding VH‐VL units found in classical Abs, and furthermore lacking the structural variability provided by the VH‐VL domain interface.^13^ The heavy chain only antibodies, from which Nbs are derived, are estimated to have evolved from classical antibodies around 25 million years ago.^10^, ^40^ Their emergence and sustained co‐existence in camelid immune systems alongside classical antibodies, is attributed to the advantageous complementary range of specificities they provide, in binding to “hidden” epitopes that are otherwise inaccessible to larger classical antibodies. 7, 9, 40 It is therefore reasonable to suggest that Nbs could have evolved different mechanisms for attaining antigen‐binding specificities comparable to Abs. In this article we use a set of bound Nb‐antigen complexes to ask how Nbs achieve a comparable range of binding specificities to classical Abs. Our dataset of co‐crystal complexes allows us to identify those aligned Nb sequence positions that make contact with the antigen, and so likely contribute to specificity determination.

We find that Nbs are notably more conserved in sequence and structure across their framework regions than classical Ab VH domain sequences. Furthermore, the Nb H1 loop is no more variable in sequence than the Ab H1 loop, and the Nb H2 loop is significantly less sequence diverse than its VH domain counterpart. Nb VHH domains are constructed from the *IGHVH* family of V genes, and our findings may support the hypothesis that this family evolved via duplication of a single *IGHV3* gene family.^10^ If VHH domains are drawn from a considerably smaller pool of germline sequences, this suggests there is less “starting diversity” across the region encoded by the V gene segment, which spans framework regions 1–3, H1 and H2. Notably, however, heavy chain antibodies have also been identified with *IGHV4* and *IGHV3* V gene origins, and so the pool of V gene sequences may not be as restricted as previously thought.^7^

Aside from their germline origins, the requirement for VHH domains to remain soluble in isolation without a partner light chain domain may provide a strong constraint on which framework sequence changes can be accepted. The finding that Nbs are more conserved in sequence and structure across their framework regions, suggests that the framework is not greatly exploited by Nbs to increase number of binding specificities that can be encoded.

How then can Nbs generate such a diverse range of binding specificities? This work identifies three potential mechanisms. The first mechanism concerns the significantly increased structural diversity exhibited by Nb H1 and H2 loops. These loops do not display greater sequence variation than their Ab counterparts, yet our structural analysis shows that they do exhibit much greater structural variability. This structural diversity may be enabled by distinct loop sequence features compared to Abs—for example, fewer Nb H1 loops contain a stabilizing F29, and Nb H2 loops use a greater proportion of small residues such as Ser and Gly, which increase structural flexibility. Regardless of how Nbs sample a greater variety of loop backbone conformations, the fact that they do makes an important contribution to their ability to achieve high specificity antigen binding.

In accordance with previous work, we find that Nb H3 loops tend to be on average longer than Ab H3 loops^7^, ^10^, ^19^, ^30^—the average length increase in our dataset is 3–4 residues. Nb H3 loops are also more divergent in both sequence and structure, occupying a greater range of positions relative to the framework. Lengthening the H3 loop provides an immediate way for Nbs to generate diversity—each extra residue increases the space of possible sequences by a factor of 20, and potentially the space of interaction specificities. Furthermore, the number of structural conformations the loops can adopt for a given sequence also increases as a function of the loop length. In addition, our dataset shows that Nbs encode around 7% more sequence diversity per amino acid residue than their Ab H3 loop counterparts. Longer H3 loops are thought to enable Nbs to bind to antigens by using fingerlike protrusions that extend into epitope cavities. Notably though, our set of Nbs also includes H3 loops that are shorter than any in the Ab dataset (Figures 5C and 6C). These findings suggest that Nbs exploit increased diversity in their H3 loops to enable them to generate the ability to bind tightly and specifically to antigens they are challenged with.

The third mechanism has not to our knowledge been previously reported in the literature. Our co‐crystal structure dataset reveals that Nb paratopes are drawn from 50 aligned sequence positions, significantly broader than the 35 positions employed by classical Ab VH domain paratopes. This is despite the fact that on average each Nb uses an average of only 2.75 additional antigen‐contacting residues compared to Ab VH domains. This suggests a novel mechanism that Nbs exploit to generate diverse binding specificities. The ability to draw paratope residues from a larger set than VH domains will promote diversity of both the shape and the physical properties of the antigen‐binding interface, enabling Nbs to use a broader range of their surface to interact with their cognate antigen through different binding modes. Perhaps surprisingly, the aligned Nb sequence positions with high contact propensity are not particularly sequence diverse. Figure 3 highlights the 50 sequence positions in our Nb alignment that are each found in > 10% of dataset paratopes; of these five are positions that would contact the VL in VH domains. For Ab VL domains the figure is 22 positions, meaning VH‐VL units have a consensus paratope of 57 positions. It appears that Nbs compensate for the missing VL domain by using a similar number of conserved paratope positions to combined VH‐VL units.

In summary, we have found that Nbs do not appear to generate specificity‐enabling diversity through increased sequence or structure diversity in the framework. It is particularly surprising that, compared to Abs, Nbs do not exploit increased sequence variation in loops H1 and H2 to compensate for the loss of the VL domain. However, Nbs do exhibit increased structural variation, in particular in the H1 loop. The Nb H3 loop is the only part of the domain with greater sequence diversity than Ab VH domains; and the majority of this increased sequence diversity is achieved through the incorporation of, on average, only 3–4 additional residues. Apparently the loss of the additional diversity that would be generated by a cognate VL domain is compensated for using a very small insertion, coupled with the freedom to sample more diverse loop conformations. This indicates that the capacity for molecular specificity in a small protein domain is much higher than we might expect based on classical Abs, suggesting that there is exciting potential for generating high affinity specific binding to a diverse range of targets using short amino acid sequences that are relatively constrained.

## Supporting information

Additional Supporting Information may be found online in the supporting information tab for this article.

Supporting Information
