# Supplementary material for: Comparative analysis of nanobody sequence and structure data
Source: Proteins. 2018 Apr 15;86(7):697–706. doi: 10.1002/prot.25497 (PMC6033041; doi:10.1002/prot.25497)
Supplement: Supplementary file 1 — Supporting Information [file PROT-86-697-s001.pdf]

Supplementary Information:

Comparative Analysis of Nanobody Sequence and Structure Data

Laura S. Mitchell[1], Lucy J. Colwell[1]

[1]Department of Chemistry, University of Cambridge, Lensfield Road, Cambridge, CB2 1EW, UK

Biotechnology and Biological Sciences Research Council, Award Number: 1501548 (L.S.M.); Marie Curie Career Integration Grant Number: 631609 (L.J.C)

FIGURE S1 Full 147-position sequence alignments of 90 Nbs. Framework regions shown in grey, H1-3 in blue, green and red. Nb alignment positions with greater than 85% gaps are shown in yellow. These positions are excluded in the reduced 126-position Nb alignment used for Figs. 2 and 3.

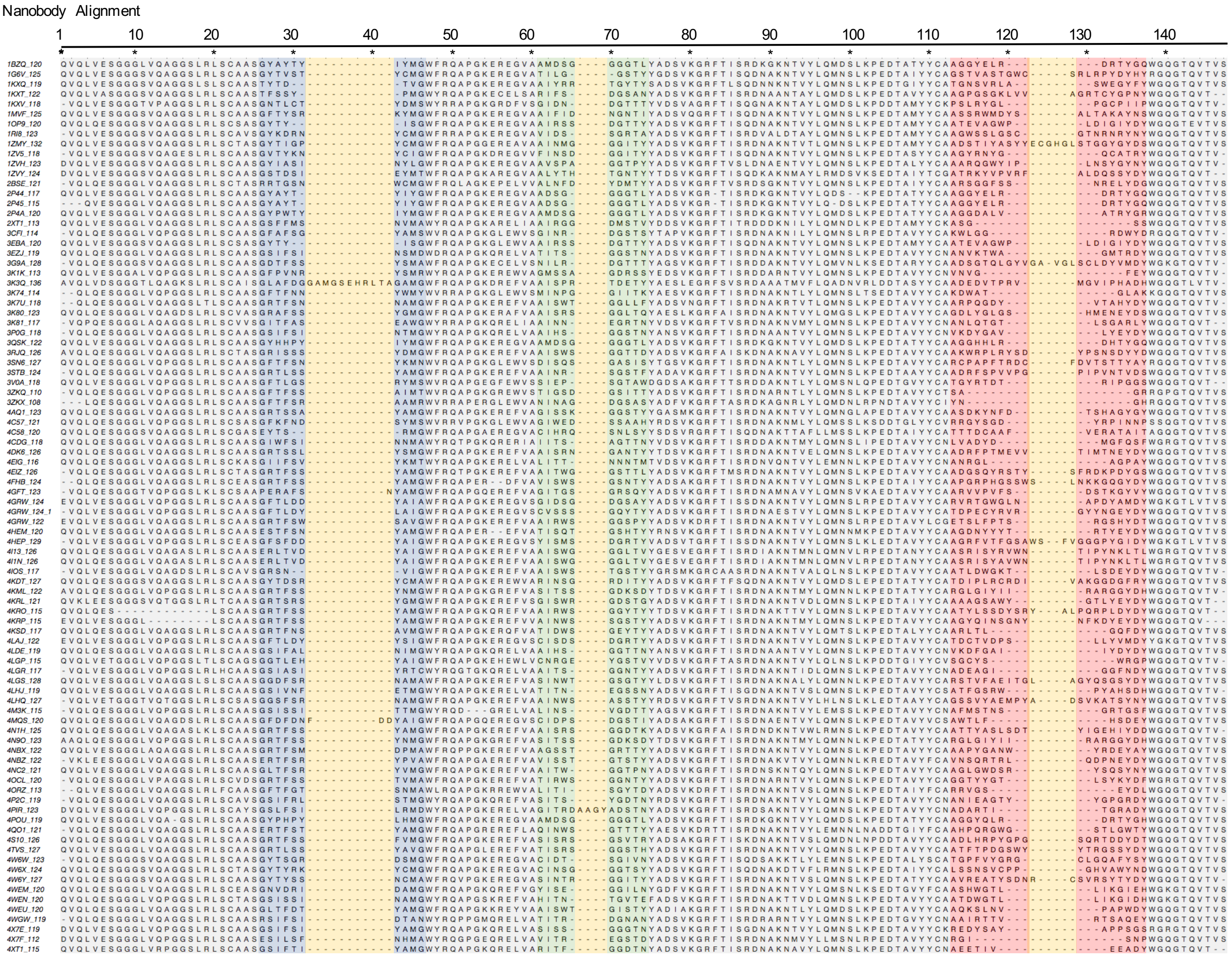

FIGURE S2 Full 147-position sequence alignments of 90 Abs. Colour scheme as above with framework regions in grey, H1-3 in blue, green and red. Positions in yellow are equivalent to those in the Nb alignment with greater than 85% gaps. These positions are excluded in the reduced 126-position Ab alignment, used for Figs. 2 and 3. Minor modifications were made to two Ab pdb files in order to fit the 147-position alignment; in 3SE8 and 4JB9 insertions occurring between 147-alignment positions 92 and 93 were deleted. These insertions are numbered 76A-76G in both pdb files, and have the sequences PDDPDWG and LYYPDRG respectively.

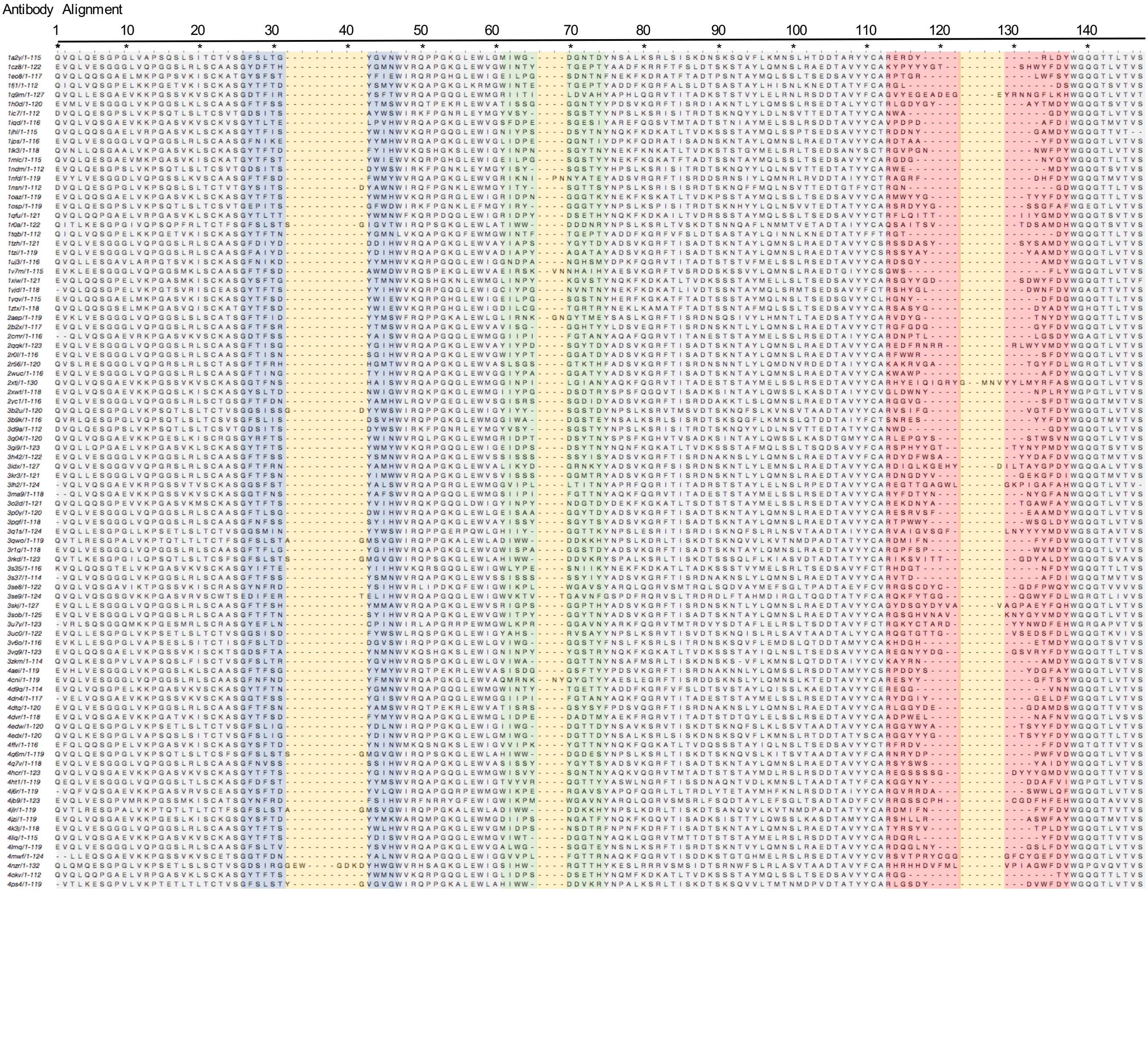

FIGURE S3 Sequence variation in sets of 1527 VHH (magenta) and 1179 VH (cyan) sequences. The number of sequences with a residue at each alignment position is plotted in dark blue, and the number of sequences with the most frequent amino acid at that position (nWT) is overlaid in magenta or cyan. The greater framework conservation across Nb frameworks noted in Fig 2 is also observed here in considerably larger sequence sets.

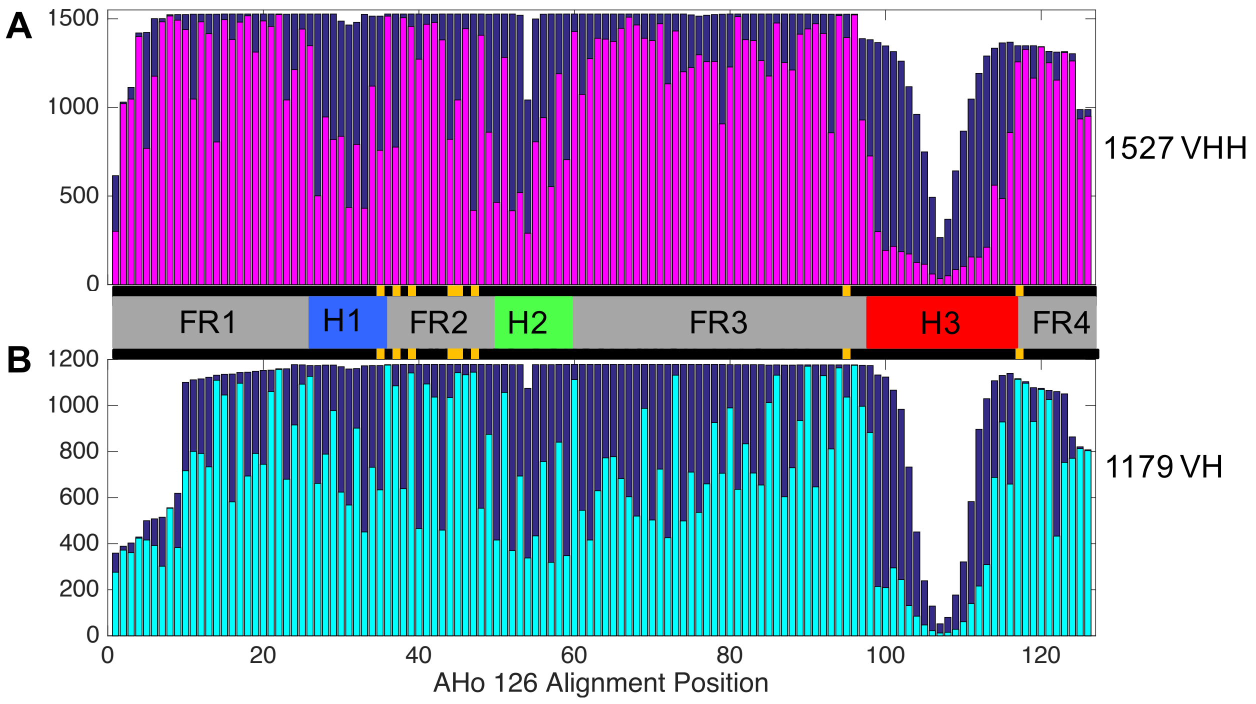

FIGURE S4 Sequence variation in sets of 90 species-unique VH (cyan) or VHH (magenta) sequences. Plots are as described in the previous figure but for sequences from A) Mouse B) Human Gamma1 C) Lama glama D) Vicugna pacos E) Camelus dromedarius.

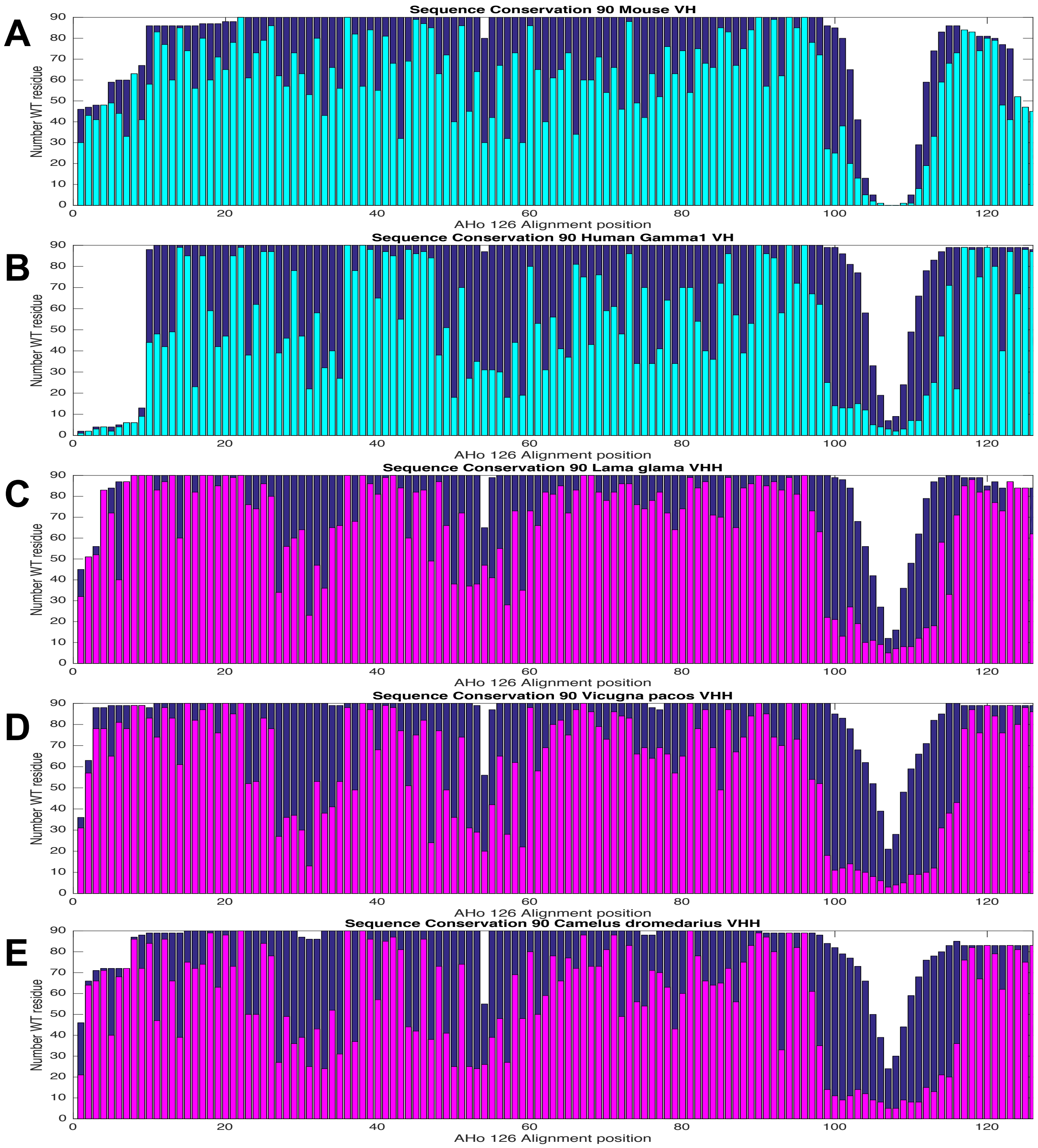

FIGURE S5 Sequence and Structural Variation of 58 Nbs in apo form. (A) Sequence logo plot (B) Structural superposition with three views.

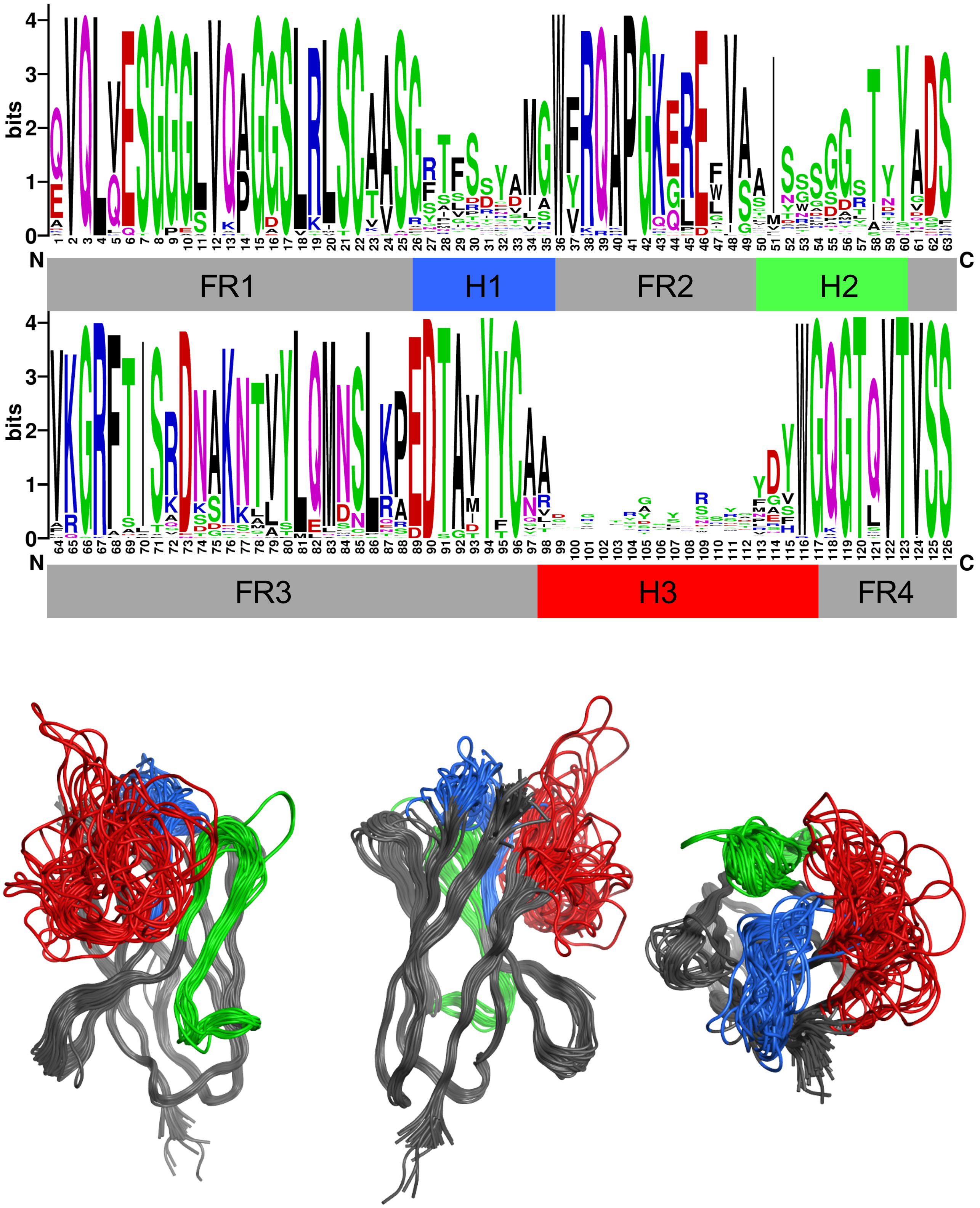

Table S1 Nanobody PDB files and Chain IDs. All structures were used as deposited in the PDB with exception to 4AQ1 and 4W6W, for which symmetry operations x,y-1,z and -y,x-y-1,z-1/3 were carried out on the respective antigen chains to obtain biologically relevant interfaces. These transformations were selected since they have the highest Complexation Significance Scores (CSS) of all crystal interfaces listed for those pdb files in the PDBePISA (Proteins, Interfaces, Structures and Assemblies) database.

Nanobodies

PDB	VHH	Ag	PDB	VHH	Ag
1BZQ	N	B	4GRW	F	CD
1G6V	K	A	4GRW	H	B
1KXQ	H	A	4HEM	E	BC
1KXT	B	A	4HEP	G	A
1KXV	C	A	4I13	B	A
1MVF	A	DE	4I1N	B	A
1OP9	A	B	4IOS	E	AB
1RI8	A	B	4KDT	A	D
1ZMY	A	L	4KML	B	A
1ZV5	A	L	4KRL	B	A
1ZVH	A	L	4KRO	B	A
1ZVY	A	B	4KRP	B	A
2BSE	E	A	4KSD	B	A
2P44	B	A	4LAJ	L	AD
2P45	B	A	4LDE	B	A
2P4A	B	A	4LGP	B	A
2XT1	B	A	4LGR	B	A
3CFI	C	B	4LGS	B	A
3EBA	A	B	4LHJ	B	A
3EZJ	B	A	4LHQ	B	A
3G9A	B	A	4M3K	B	A
3K1K	C	A	4MQS	B	A
3K3Q	A	B	4N1H	B	A
3K74	B	A	4N9O	B	A
3K7U	A	C	4NBX	B	A
3K80	A	C	4NBZ	B	A
3K81	A	D	4NC2	B	A
3P0G	B	A	4OCL	C	B
3QSK	B	A	4ORZ	C	B
3RJQ	B	A	4P2C	H	AC
3SN6	N	AB	4PIR	G	AB
3STB	A	D	4POU	B	A
3V0A	C	A	4QO1	A	B
3ZKQ	D	A	4S10	A	C
3ZKX	C	A	4TVS	a	A
4AQ1	D	C	4W6W	B	A
4C57	C	A	4W6X	B	A
4C58	B	A	4W6Y	B	A
4CDG	C	A	4WEM	B	A
4DK6	A	D	4WEN	B	A
4EIG	B	A	4WEU	E	A
4EIZ	D	A	4WGW	B	A
4FHB	D	A	4X7E	C	A
4GFT	B	A	4X7F	C	A
4GRW	E	CD	4XT1	C	A

Table S2 Antibody PDB files and Chain IDs.

Antibodies

PDB	VH VL	Ag	PDB	VH VL	Ag
1A2Y	BA	C	3KR3	HL	D
1CZ8	YX	V	3LH2	JN	U
1EO8	HL	A	3MA9	HL	A
1FJ1	DC	E	3O2D	HL	A
1G9M	HL	G	3P0Y	HL	A
1H0D	BA	C	3PGF	HL	A
1IC7	HL	Y	3Q1S	HL	I
1IQD	BA	C	3QWO	AB	C
1JHL	HL	A	3R1G	HL	B
1JPS	HL	T	3RKD	HL	A
1LK3	IM	B	3S35	HL	X
1MLC	DC	F	3S37	HL	X
1NDM	BA	C	3SE8	HL	G
1NFD	FE	B	3SE9	HL	G
1NSN	HL	S	3SKJ	HL	E
1OAZ	HL	A	3SOB	HL	B
1OSP	HL	O	3U7Y	HL	G
1QFU	HL	A	3UC0	IM	B
1R0A	HL	B	3V6O	CE	A
1TQB	BC	A	3VG9	CB	A
1TZH	BA	W	3ZKM	HL	A
1TZI	BA	V	4AEI	HL	A
1UJ3	BA	C	4CNI	AB	D
1V7M	IM	X	4D9Q	ED	B
1XIW	DC	A	4DN4	HL	M
1YJD	HL	C	4DTG	HL	K
1YQV	HL	Y	4DVR	HL	G
1ZTX	HL	E	4EDW	HL	V
2AEP	HL	A	4EDX	HL	V
2B2X	IM	B	4FFV	HL	A
2CMR	HL	A	4G6M	HL	A
2QQK	HL	A	4G7V	HL	S
2R0L	HL	A	4HCR	HL	A
2R56	IM	B	4HT1	HL	T
2WUC	HL	A	4J6R	HL	G
2XTJ	DB	A	4JB9	HL	G
2XWT	AB	C	4JLR	HL	S
2YC1	AB	C	4JZJ	HL	C
3B2U	JK	I	4K3J	HL	B
3B9K	HL	B	4LIQ	HL	E
3D9A	HL	C	4LMQ	EI	F
3G04	BA	C	4MWF	AB	D
3GI9	HL	C	4NZR	HL	M
3H42	HL	BA	4OKV	AB	F
3IDX	HL	G	4PS4	HL	A

TABLE S3 Apo form Nanobody PDB files and Chain IDs.

Apo form Nanobodies			
**PDB**	**VHH**	**PDB**	**VHH**
1F2X	L	4KRN	A
1HCV	A	4M3J	A
1OHQ	B	4PFE	B
1SHM	A	4POY	A
1SJX	A	4PPT	A
1T2J	A	4QGY	B
1U0Q	A	4QLR	B
1YC7	B	4S11	A
1YC8	B	4TYU	B
1YZZ	B	4U7S	B
2X1O	A	4ZG1	A
2XA3	A	5DA4	B
2XXC	B	5DXW	A
3B9V	A	5E7B	A
3DWT	H	5FWO	A
3EAK	A	5H8D	A
3LN9	A	5HDO	A
3QXU	C	5I0Z	B
3QXW	B	5IVO	A
3QYC	B	5JMR	A
3R0M	A	5LMJ	A
3TPK	A	5LMW	A
3ZHD	A	5LZ0	B
3ZHK	A	5NLU	A
3ZHL	A	5NLW	A
4AQ1	B	5NM0	A
4B41	A	5NML	E
4B5E	A	5SV4	A
4IDL	A	5TP3	B

TABLE S4 Alignment position to AHo numbering index table. The nanobody consensus sequence is listed in the third column for reference – the capitalisation threshold is 90%, and '+' indicates positions with multiple modal residues. H1-3 loop positions used in this study are coloured blue, green and red.

AHo 126	AHo numbering	Nb Consensus
1	1	q
2	2	V
3	3	Q
4	4	L
5	5	q
6	6	E
7	7	S
8	9	G
9	10	G
10	11	G
11	12	l
12	13	V
13	14	Q
14	15	a
15	16	G
16	17	g
17	18	S
18	19	L
19	20	R
20	21	L
21	22	S
22	23	C
23	24	a
24	25	A
25	26	S
26	27	g
27	29	r
28	30	t
29	31	f
30	32	s
31	33	s
32	39	y
33	40	a
34	41	m
35	42	g
36	43	W
37	44	f
38	45	R
39	46	Q
40	47	a
41	48	P
42	49	G
43	50	k
44	51	e
45	52	r
46	53	E
47	54	f
48	55	V
49	56	a
50	57	a
51	58	i
52	59	s
53	60	s
54	61	s
55	65	g
56	66	g
57	67	s
58	68	t
59	69	y
60	70	Y
61	71	a
62	72	d
63	73	S
64	74	V
65	75	K
66	76	G
67	77	R
68	78	F
69	79	T
70	80	i
71	81	S
72	82	r
73	83	D
74	84	n
75	85	a
76	86	k
77	87	n
78	88	t
79	89	v
80	90	y
81	91	L
82	92	q
83	93	M
84	94	n
85	95	s
86	96	L
87	97	k
88	98	p
89	99	E
90	100	D
91	101	T
92	102	A
93	103	v
94	104	Y
95	105	Y
96	106	C
97	107	a
98	108	a
99	109	g
100	110	+
101	111	+
102	112	s
103	113	+
104	114	r
105	115	s
106	116	+
107	117	l
108	130	+
109	131	l
110	132	r
111	133	g
112	134	y
113	135	g
114	136	y
115	137	d
116	138	y
117	139	w
118	140	G
119	141	q
120	142	G
121	143	T
122	144	Q
123	145	V
124	146	T
125	147	V
126	148	s

TABLE S5 Alignment positions colour-coded according to extent of difference in conservation between Nb and Ab alignment. The first two columns of this table contain the numbering for both the AHo length 126 sequence alignment, and the extended 147 sequence alignment that includes the loop centres. The next five columns contain statistics for five additional AHo Ab alignments, constructed by sampling sets of 90 Ab sequences from the larger set of 191 co-crystal structures (columns Ab_A to Ab_E). For each of these alignments, we then calculated the percentage of wild type amino acid residues at each aligned sequence position (percentage nWT), as reported in these columns. The mean and standard deviation are then reported in the final two columns of the table. The idea was to obtain a bootstrapped sampling of the statistics of the Ab alignment. We then compared these statistics to the percentage of wild type amino acid residues at each aligned sequence position of the Nb alignment. Those positions highlighted in purple in the first two columns are significantly more conserved in Nbs ($nWT > \mu + 2\sigma$), while those highlighted in orange are significantly less conserved in Nbs ($nWT < \mu - 2\sigma$). Those positions that are not highlighted are approximately equally conserved across Nbs and Abs ($\mu - 2\sigma <$ $nWT > \mu + 2\sigma$).

	Position	Nb	Ab	Ab_A	Ab_B	Ab_C	Ab_D	Ab_E	Ab Mean	Ab SD
FR1	1	**78.3**	50.6	46.6	51.3	53.1	47.9	54.4	**50.7**	2.98
	2	**98.8**	92.0	98.9	92.0	94.4	94.3	94.3	**94.3**	2.50
	3	**97.7**	80.0	80.0	82.2	87.8	88.8	88.8	**84.6**	4.31
	4	**97.8**	96.7	98.9	100.0	97.8	97.8	97.8	**98.1**	1.15
	5	**51.1**	50.0	41.1	45.6	47.8	42.2	43.3	**45.0**	3.42
	6	**97.8**	58.9	53.3	57.8	51.1	54.4	56.7	**55.4**	2.93
	7	**97.8**	95.6	95.6	94.4	95.6	95.6	93.3	**95.0**	0.93
	8	**100.0**	98.9	100.0	100.0	100.0	100.0	100.0	**99.8**	0.45
	9	**100.0**	34.4	37.8	34.4	33.3	36.7	37.8	**35.7**	1.91
	10	**98.9**	47.8	43.3	44.4	45.6	43.3	43.3	**44.6**	1.78
	11	**75.3**	72.2	77.8	76.7	76.7	75.6	82.2	**76.9**	3.25
	12	**96.6**	66.7	72.2	70.0	74.4	67.8	77.8	**71.5**	4.20
	13	**96.6**	57.8	53.3	53.3	55.6	61.1	65.6	**57.8**	4.82
	14	**72.7**	98.9	100.0	100.0	100.0	98.9	98.9	**99.4**	0.61
	15	**100.0**	76.7	74.4	78.9	80.0	74.4	71.1	**75.9**	3.27
	16	**90.9**	30.0	28.9	31.1	31.1	32.2	30.0	**30.6**	1.17
	17	**100.0**	77.8	80.0	84.4	86.7	83.3	78.9	**81.9**	3.49
	18	**100.0**	56.7	53.3	54.4	56.7	52.2	58.9	**55.4**	2.48
	19	**93.2**	41.1	43.3	50.0	44.4	43.3	46.7	**44.8**	3.12
	20	**100.0**	58.9	57.8	55.6	60.0	61.1	62.2	**59.3**	2.40
	21	**96.7**	77.8	76.7	80.0	82.2	78.9	74.4	**78.3**	2.70
	22	**100.0**	98.9	100.0	100.0	100.0	100.0	100.0	**99.8**	0.45
	23	**78.9**	35.6	33.3	38.9	33.3	35.6	35.6	**35.4**	2.04
	24	**91.1**	66.7	62.2	68.9	70.0	67.8	61.1	**66.1**	3.63
	25	**98.9**	90.0	92.2	92.2	94.4	88.9	85.6	**90.6**	3.12
H1	26	**86.7**	97.8	97.8	100.0	98.9	100.0	100.0	**99.1**	1.09
	27	**28.9**	44.4	47.8	54.4	47.8	44.4	43.3	**47.0**	4.08
	28	**55.6**	50.0	43.3	52.2	51.1	46.7	46.7	**48.3**	3.35
	29	**48.9**	62.2	60.0	58.9	64.4	58.9	53.3	**59.6**	3.76
	30	**55.3**	35.6	36.7	41.1	43.3	36.7	43.3	**39.4**	3.57
	31	**26.8**	29.2	26.7	30.0	27.8	32.2	35.6	**30.2**	3.23
	32	**53.6**	64.4	52.2	57.8	66.7	58.9	60.0	**60.0**	5.12
	33	**34.4**	20.0	26.7	21.1	24.4	23.3	21.1	**22.8**	2.51
	34	**75.6**	35.6	36.7	35.6	40.0	35.6	34.4	**36.3**	1.95
	35	**70.0**	28.9	40.0	34.4	40.0	32.2	31.1	**34.4**	4.66
FR2	36	**100.0**	97.8	98.9	98.9	100.0	100.0	100.0	**99.3**	0.91
	37	**68.9**	72.2	74.4	77.8	77.8	75.6	72.2	**75.0**	2.51
	38	**100.0**	80.0	74.4	73.3	74.4	74.4	73.3	**75.0**	2.51
	39	**93.3**	90.0	92.2	97.8	93.3	93.3	90.0	**92.8**	2.88
	40	**88.9**	45.6	42.2	43.3	44.4	44.4	37.8	**43.0**	2.78
	41	**97.8**	87.8	86.5	88.9	89.9	93.3	90.0	**89.4**	2.31
	42	**96.6**	91.1	86.7	87.8	85.6	90.0	86.7	**88.0**	2.16
	43	**84.3**	61.1	60.0	65.6	61.1	52.2	56.7	**59.4**	4.54
	44	**63.6**	77.8	73.3	77.8	81.1	80.0	70.0	**76.7**	4.22
	45	**87.5**	92.2	93.3	96.7	95.6	95.6	96.7	**95.0**	1.83
	46	**95.6**	95.6	98.9	96.7	98.9	98.9	96.7	**97.6**	1.48
	47	**38.9**	93.3	95.6	97.8	96.7	95.6	90.0	**94.8**	2.78
	48	**93.3**	31.1	33.3	31.1	33.3	30.0	30.0	**31.5**	1.52
	49	**80.0**	72.2	72.2	67.8	72.2	76.7	76.7	**73.0**	3.35
H2	50	**41.1**	15.6	15.6	13.3	14.4	12.2	16.7	**14.6**	1.64
	51	**85.6**	82.2	85.6	92.2	86.7	91.1	90.0	**88.0**	3.81
	52	**31.1**	18.9	17.8	17.8	21.1	16.7	21.1	**18.9**	1.86
	53	**31.1**	46.1	48.9	48.9	47.8	48.9	48.9	**48.2**	1.15
	54	**39.3**	19.7	19.4	20.0	27.1	23.5	27.0	**22.8**	3.63
	55	**56.2**	23.3	24.4	18.9	26.7	23.3	28.9	**24.3**	3.40
	56	**67.8**	64.4	67.8	67.8	66.7	70.0	74.4	**68.5**	3.42
	57	**27.8**	14.4	14.4	16.7	16.7	18.9	20.0	**16.9**	2.27
	58	**77.8**	65.6	64.4	68.9	70.0	61.1	76.7	**67.8**	5.40
	59	**40.0**	20.0	20.0	16.7	20.0	22.2	22.2	**20.2**	2.04
	60	**94.4**	91.1	90.0	95.6	91.1	93.3	94.4	**92.6**	2.18
	61	**70.0**	44.4	42.2	38.9	40.0	40.0	44.4	**41.7**	2.41
	62	**83.3**	27.8	26.7	31.1	28.9	28.9	28.9	**28.7**	1.48
	63	**94.4**	44.4	40.0	40.0	40.0	40.0	42.2	**41.1**	1.86
FR3	64	**90.0**	41.1	46.7	41.1	46.7	45.6	41.1	**43.7**	2.87
	65	**91.1**	68.9	65.6	61.1	62.2	60.0	67.8	**64.3**	3.68
	66	**96.7**	60.0	50.0	63.3	58.9	54.4	51.1	**56.3**	5.29
	67	**100.0**	76.7	73.3	73.3	68.9	72.2	71.1	**72.6**	2.60
	68	**97.7**	35.6	30.0	34.4	34.4	27.8	30.0	**32.0**	3.18
	69	**94.4**	78.9	81.1	86.7	82.2	81.1	76.7	**81.1**	3.37
	70	**86.7**	62.2	62.2	68.9	61.1	65.6	68.9	**64.8**	3.49
	71	**98.9**	58.9	50.0	56.7	55.6	52.2	50.0	**53.9**	3.70
	72	**77.8**	33.3	33.3	37.8	33.3	34.4	33.3	**34.3**	1.78
	73	**98.9**	95.6	97.8	97.8	96.7	96.7	97.8	**97.0**	0.91
	74	**76.7**	47.8	46.7	47.8	43.3	51.1	53.3	**48.3**	3.50
	75	**86.7**	85.6	88.9	90.0	90.0	91.1	85.6	**88.5**	2.40
	76	**83.3**	46.7	45.6	44.4	45.6	47.8	48.9	**46.5**	1.64
	77	**88.9**	52.8	47.8	54.4	53.3	53.3	57.8	**53.2**	3.23
	78	**86.7**	65.6	65.6	70.0	73.3	67.8	64.4	**67.8**	3.37
	79	**72.2**	51.1	51.1	53.3	52.2	51.1	46.7	**50.9**	2.27
	80	**81.1**	72.2	70.0	67.8	70.0	67.8	72.2	**70.0**	1.99
	81	**100.0**	66.7	63.3	65.6	67.8	64.4	67.8	**65.9**	1.81
	82	**88.9**	52.2	50.0	54.4	60.0	55.6	61.1	**55.6**	4.33
	83	**94.4**	42.2	51.1	44.4	44.4	55.6	54.4	**48.7**	5.73
	84	**74.4**	46.7	43.3	46.7	43.3	42.2	46.7	**44.8**	2.07
	85	**79.8**	78.9	81.1	82.2	77.8	85.6	85.6	**81.9**	3.27
	86	**95.5**	77.8	77.8	85.6	81.1	76.7	76.7	**79.3**	3.49
	87	**83.3**	46.7	41.1	44.4	41.1	44.4	43.3	**43.5**	2.16
	88	**87.8**	38.9	42.2	44.4	40.0	43.3	43.3	**42.0**	2.16
	89	**92.2**	64.4	68.9	67.8	65.6	67.8	72.2	**67.8**	2.72
	90	**100.0**	98.9	100.0	100.0	100.0	100.0	100.0	**99.8**	0.45
	91	**100.0**	81.1	75.6	78.9	80.0	80.0	78.9	**79.1**	1.91
	92	**92.2**	94.4	97.8	97.8	98.9	97.8	97.8	**97.4**	1.52
	93	**56.7**	61.1	58.9	65.6	64.4	70.0	58.9	**63.1**	4.36
	94	**100.0**	98.9	97.8	97.8	98.9	98.9	97.8	**98.3**	0.61
	95	**91.1**	80.0	83.3	83.3	83.3	82.2	86.7	**83.1**	2.16
	96	**100.0**	97.8	100.0	98.9	100.0	100.0	100.0	**99.4**	0.93
	97	**72.2**	73.3	70.0	72.2	68.9	80.0	73.3	**73.0**	3.89
H3	98	**63.3**	80.0	72.2	81.1	76.7	73.3	77.8	**76.9**	3.54
	99	**23.3**	17.8	15.6	16.7	14.4	16.7	16.7	**16.3**	1.15
	100	**11.4**	18.9	20.0	18.9	15.6	16.9	17.8	**18.0**	1.61
	101	**14.0**	15.9	22.9	16.7	17.0	23.5	22.2	**19.7**	3.53
	102	**15.3**	22.1	19.0	15.4	18.8	24.7	22.4	**20.4**	3.31
	103	**12.8**	28.3	23.6	30.9	26.7	34.5	34.7	**29.8**	4.42
	104	**13.5**	18.4	18.8	25.9	21.2	27.3	15.6	**21.2**	4.56
	105	**23.5**	33.3	27.8	26.7	19.0	16.7	18.8	**23.7**	6.54
	106	**16.1**	20.0	33.3	25.0	27.3	25.0	25.0	**25.9**	4.34
	107	**21.7**	28.6	25.0	50.0	50.0	33.3	16.7	**33.9**	13.59
	108	**17.9**	22.2	25.0	37.5	22.2	25.0	28.6	**26.8**	5.76
	109	**17.1**	12.5	18.2	18.2	14.3	15.8	16.7	**15.9**	2.24
	110	**13.3**	20.7	23.1	15.8	20.0	28.6	23.8	**22.0**	4.29
	111	**13.0**	17.0	17.5	18.2	17.8	17.1	13.5	**16.8**	1.69
	112	**18.3**	23.5	29.6	24.2	25.3	32.8	34.4	**28.3**	4.63
	113	**20.0**	21.0	27.7	26.8	28.7	28.6	24.7	**26.3**	2.97
	114	**56.3**	55.8	53.4	51.8	46.7	50.6	51.2	**51.6**	3.05
	115	**47.8**	70.0	78.9	67.8	75.6	76.7	81.1	**75.0**	5.15
	116	**54.4**	55.6	55.6	62.2	54.4	53.3	56.7	**56.3**	3.12
FR4	117	**88.9**	98.9	100.0	100.0	100.0	100.0	100.0	**99.8**	0.45
	118	**98.9**	97.8	98.9	98.9	97.8	98.9	100.0	**98.7**	0.84
	119	**86.7**	85.6	83.3	88.9	86.7	81.1	82.2	**84.6**	2.93
	120	**100.0**	98.9	100.0	100.0	100.0	100.0	100.0	**99.8**	0.45
	121	**100.0**	95.6	98.9	97.8	97.8	94.4	98.9	**97.2**	1.83
	122	**95.6**	51.1	42.2	47.8	44.4	46.7	37.8	**45.0**	4.65
	123	**100.0**	83.3	85.6	83.3	90.0	86.7	82.2	**85.2**	2.87
	124	**100.0**	92.2	90.0	96.7	92.2	97.8	95.6	**94.1**	3.04
	125	**100.0**	98.9	100.0	98.9	100.0	98.9	100.0	**99.4**	0.61
	126	**100.0**	97.7	100.0	98.9	98.9	98.9	98.9	**98.9**	0.73
